# “You are not a man”: a multi‐method study of trans stigma and risk of HIV and sexually transmitted infections among trans men in Uganda

**DOI:** 10.1002/jia2.25860

**Published:** 2021-12-29

**Authors:** Andrew Mujugira, Vicent Kasiita, Monica Bagaya, Agnes Nakyanzi, Felix Bambia, Oliva Nampewo, Brenda Kamusiime, Jackson Mugisha, Alisaati Nalumansi, Collin C. Twesigye, Timothy R. Muwonge, Jared M. Baeten, Monique A. Wyatt, Alexander C. Tsai, Norma C. Ware, Jessica E. Haberer

**Affiliations:** ^1^ Infectious Diseases Institute Makerere University Kampala Uganda; ^2^ Department of Epidemiology and Biostatistics School of Public Health Makerere University Kampala Uganda; ^3^ Department of Global Health University of Washington Seattle Washington USA; ^4^ Department of Global Health and Social Medicine Harvard Medical School Boston Massachusetts USA; ^5^ Harvard Global Cambridge Massachusetts USA; ^6^ Massachusetts General Hospital and Harvard Medical School Boston Massachusetts USA

**Keywords:** trans men, HIV, STI, stigma, Africa

## Abstract

**Introduction:**

Transgender (trans) men in sub‐Saharan Africa are a hidden and vulnerable population who may engage in sex work due to socio‐economic exclusion and lack of alternative employment opportunities. Little is known about HIV and sexually transmitted infection (STI) risk among trans men in this setting. We conducted a multi‐method study to characterize HIV/STI risk among trans men in Uganda.

**Methods:**

Between January and October 2020, we enrolled 50 trans men into a cross‐sectional study through snowball sampling. Data were collected on socio‐demographic characteristics, sexual practices and depression. We conducted 20 qualitative interviews to explore: (1) descriptions of sexual practices that could increase HIV/STI exposure; (2) experiences of accessing public healthcare facilities; (3) perceptions of HIV or STI testing; (4) HIV and STI service delivery; and (5) drug and alcohol use. We used an inductive content analytic approach centring on descriptive category development to analyse the data.

**Results:**

The median age was 25 years (interquartile range 23–28). The prevalence of HIV, syphilis and hepatitis B was 4%, 6% and 8%, respectively. We observed multiple levels of intersecting individual, interpersonal and structural stigmas. (1) Trans men reported transphobic rape motivated by interpersonal stigma that was psychologically traumatizing to the survivor. The resultant stigma and shame hindered healthcare access. (2) Structural stigma and economic vulnerability led to sex work, which increased the risk of HIV and other STIs. Sex work stigma further compounded vulnerability. (3) Individualized stigma led to fear of disclosure of gender identity and HIV status. Concealment was used as a form of stigma management. (4) Multiple levels of stigma hampered access to healthcare services. Preference for trans‐friendly care was motivated by stigma avoidance in public facilities. Overall, the lived experiences of trans men highlight the intertwined relationship between stigma and sexual health.

**Conclusions:**

In this sample from Uganda, trans men experienced stigma at multiple levels, highlighting the need for gender‐sensitive healthcare delivery. Stigma reduction interventions, including provider training, non‐discrimination policies, support groups and stigma counselling, could strengthen uptake and utilization of prevention services by this marginalized population.

## 1 INTRODUCTION

Transgender (trans) men, individuals who are men, male or other masculine identities and were assigned female sex at birth, are often thought to be at low risk of human immunodeficiency virus (HIV) acquisition because of the assumption that they have sex only with cisgender women, which is considered low‐risk sexual activity [1]. Emerging data suggest that trans men face vulnerability to HIV through condomless vaginal and/or anal sex with cisgender men, having multiple sexual partners, not knowing the HIV positive or unknown HIV status of these partners and having sex while under the influence of alcohol [2, 3, 4]. HIV vulnerability is exacerbated by social marginalization, transphobia, violence and homophobic rape [5, 6, 7, 8]. Few data are available on HIV prevalence among trans men globally. HIV prevalence among trans men in the United States (3.2%) is 10‐fold higher than in the general population (0.3%) [4, 9], but this estimate is based on few studies with small sample sizes [10, 11, 12].

Other HIV risk factors centre around sex work and sexually transmitted infections (STIs) among trans men. Sex work is common; 13% of trans men in the United States report engaging in sex work, similar to proportions of trans women [4]. Trans men who engage in sex work typically have sex with cisgender men [13]. Globally, HIV prevalence among trans women sex workers is twice as high as the HIV prevalence among cisgender men who have sex with cisgender men [14], but few data are available for trans men sex workers, particularly in sub‐Saharan Africa [15]. In one study among sex workers in Zimbabwe, HIV prevalence was 38.1% in trans men compared with 37.6% in trans women, 36.5% in cisgender women and 28.2% in cisgender men [16]. Additionally, little is known about STI prevalence in this population. The prevalence of syphilis, gonorrhoeae and chlamydia, respectively, ranged from 0% to 4.2%, 0% to 10.5% and 1.2% to 11.1% in six studies of trans men [11, 17, 18, 19, 20, 21].

Trans men have been largely ignored in HIV and STI research globally [13, 22, 23], despite their greater vulnerability compared with the general population. No published studies have evaluated HIV and STI risk among trans men in sub‐Saharan Africa. We conducted a multi‐method study to characterize HIV and STI risk, prevention needs and sexual decision making among trans men in Uganda.

## 2 METHODS

### 2.1 Study design and participants

We conducted a cross‐sectional study among trans men in Uganda from January to October 2020. The study was conducted at the Makerere University Infectious Diseases Institute research clinic in Kasangati, Uganda. Since 2018, five HIV prevention research studies among trans women and trans men have been implemented at the research facility, which is easily accessible by public transportation [24]. The research team utilizes a trans‐inclusive approach incorporating community engagement, provider sensitivity training, peer support, inclusion of trans people in research design and implementation, and as co‐investigators and members of the community advisory group, plus quarterly consultative meetings for trans people, peers and research staff. Sexual and gender minorities report that the research clinic is inclusive and welcoming, and that they feel safe while there [25].

We asked about gender identity using a two‐step approach: (1) assessment of sex assignment at birth and (2) current self‐reported gender identity [26]. Inclusion criteria were female sex assigned at birth, current identification as male and being aged 18 years or older. Individuals aged 14–17 years who qualified as a mature or emancipated minor due to having an STI, drug or alcohol dependency, or catering for one's own livelihood were also eligible to participate [27]. Exclusion criteria were any clinically significant or chronic medical condition or mental illness, which precluded provision of informed consent. We recruited trans men through snowball sampling, a recruitment strategy used to reach hidden populations [28]. Initial peer recruiters were identified by transgender‐led community organizations who were asked to recruit from their social networks. Participants enrolled in the study were also asked to recruit from their social networks across the country. We recruited participants living with and without HIV to characterize factors, which influenced the uptake and utilization of HIV/STI services. Peer recruiters were compensated with 10,000 Uganda Shillings (UGX; $2.72) for each referred participant subsequently enrolled.

### 2.2 Study procedures

Prior to study initiation, research staff received gender sensitivity training, including from trans men peer educators [29]. Quantitative data were collected using Research Electronic Data Capture (REDCap) electronic data capture tools hosted at the University of Washington [30]. Questionnaires collected data on socio‐demographic characteristics (i.e. age, gender identity, education, income sources, marital status, alcohol and drug use) and sexual behaviours (i.e. vaginal, anal and oral sex, condom use, prior history of STIs, HIV and STI testing and sex work). Choice and wording of questions on sensitive sexual practices reflected recommendations from the community. We used validated socio‐behavioural scales for stigma (HIV stigma scale [31]) and problematic alcohol use (RAPS‐4 scale [32]). All participants were screened for depression using the Patient Health Questionnaire (PHQ‐2 and PHQ‐9) [33]. The PHQ‐9 was completed if a participant scored ≥3 on the PHQ‐2; those with PHQ‐9 scores ≥10 were escorted to the Infectious Diseases Institute mental health clinic, if desired. Participants were provided with free HIV and STI screening and treatment. Those who tested HIV positive, and were not already receiving care, were actively linked to HIV treatment at a facility of their choice.

We invited a purposively sampled subset of participants with a range of relevant life experiences (self‐report of stigma, discrimination, harassment and/or arrest for being transgender; multiple sexual partners; hormonal therapy use; and PrEP use) to take part in a single in‐person qualitative interview after administration of the quantitative survey, usually at the same clinic visit. Interview topics included: (1) descriptions of sexual practices that may increase HIV/STI exposure, including sex work; (2) experiences of accessing public healthcare facilities; (3) perceptions of HIV or STI testing; (4) perspectives on how HIV and STI services should be provided to trans men; and (5) drug and alcohol use. Interviews were about 1 hour long, were conducted by trained cisgender women and men proficient in Luganda (local language) and English and took place at the study clinic in a private place where conversations could not be overheard. Interviews were audio‐recorded with permission, transcribed and translated into English by the interviewer. We performed quality checks for each transcript to identify and correct errors. Participants were compensated with UGX 30,000 ($8.16) in accordance with local research ethics committee guidance.

Participants received point‐of‐care testing for HIV and syphilis (SD Bioline HIV/Syphilis Duo test; Abbott, Abbott Park, IL, USA), hepatitis B virus (HBV) (Pal^®^ HBsAg rapid test; Healgan Scientific, Houston, TX, USA) and pregnancy (QuickVue hCG urine test; Quidel Corporation, San Diego, CA, USA) according to national guidelines (N.B., funding was not available for testing other STIs). All participants provided written informed consent in English or Luganda. The study received ethical approval from the Makerere University School of Public Health (Higher Degrees Research Ethics Committee, protocol 500), Uganda National Council for Science and Technology (SS 4467) and MassGeneral Brigham Human Research Committee (protocol 2017P001951).

### 2.3 Data analysis

Quantitative data were analysed descriptively using Stata 14 (StataCorp, College Station, TX, USA). We used an inductive, content analytic approach to analyse the qualitative data [34]. Interview transcripts were repeatedly reviewed for content on sexual health experiences. We performed open coding to identify specific sections of text by delineating and provisionally labelling relevant content. Provisional labels were then defined into code names and assembled into a codebook. We coded the data using Dedoose software (version 9.0, www.dedoose.com, SCRC, Hermosa Beach, CA, USA) to organize the coding process. On completion of the coding process, we used queries to sort the data and identify concepts corresponding to stigma experiences. Content categories were developed from emerging themes. Each category consists of a descriptive label, elaborative text and interview quotes illustrating the concept. Categories are described in the Qualitative Results, below. The primary themes identified in the data are represented in these categories; saturation was achieved. We used the Consolidated Criteria for Reporting Qualitative Studies checklist for reporting study findings [35].

## 3 RESULTS

### 3.1 Participant characteristics

We screened 52 people and enrolled 50 trans men (two were ineligible because they identified as women). The median age was 25 years (interquartile range [IQR] 23–28) and the median duration of education was 13 years of schooling (IQR 12–15) (Table 1). Most (19; 62%) were self‐employed, 10 (20%) were unemployed and none reported sex work as their primary occupation. The median monthly personal income was UGX 300,000 ($77.70). Two‐thirds (68%) had a romantic partner and most of these partners (42; 84%) were cisgender women. Participants reported attraction to women (64%), attraction to women and men (26%) and attraction regardless of gender (8%). They reported a median of 4 sex acts (IQR 0–16) in the prior 3 months. Most (36; 72%) had more than one partner, of which 42% (15/36) reported new sexual partners. Of the 50 trans men, 43 (86%) reported having vaginal sex, of whom 23 (46%) reported knowing the HIV status of their sexual partner; 91% (21/23) said their partner was HIV negative. Seventeen trans men (34%) ever had sex with cisgender men. Five (10%) reported anal sex. The proportion currently using alcohol and tobacco was 60% and 22%, respectively. Twenty‐four percent (12/50) were currently using recreational drugs, commonly shisha (flavoured tobacco) (67%), marijuana (42%), kuber (smokeless tobacco) (25%) and khat (*Catha edulis;* plant leaves chewed for stimulant and euphoric effects) (25%). None reported cocaine or heroin use.

**Table 1:** Participant characteristics

Variable	*N* (%) or median (IQR)
Age	25.2 (23.2–27.9)
Education (completed years)	13 (12–15)
Occupation	
Self‐employed	19 (62)
Restaurant/bar	4 (8)
Sex work	0 (0)
Unemployed	10 (20)
Other specify	18 (36)
Monthly income (UGX; *n* = 31)	300,000 (150,000–500,000)
Current partnership status	
Divorced/separated	2 (4)
Has a partner	34 (68)
Other(specify)	2 (4)
Single with no partner	12 (24)
Sexual partners, prior 3 months	
0	14 (28)
1	28 (56)
2	6 (12)
3	1 (2)
4	1 (2)
New sexual partners (*n* = 36)	
Yes	15 (42)
No	21 (58)
Non‐consensual sex, prior month (*n* = 13)	
Yes	2 (15)
No	11 (85)
Ever been harassed by law enforcement for being transgender	
Yes	17 (34)
No	33 (66)
Ever been incarcerated	
Yes	15 (30)
No	35 (70)
If yes, arrest related to being transgender (*n* = 15)	
Yes	12 (80)
No	3 (20)
Ever used hormone therapy or cross‐sex hormones	
Yes	9 (18)
No	34 (68)
Missing	7 (14)
If yes, currently taking hormone therapy (*n* = 9)	
Yes	6 (67)
No	3 (33)
If yes, where do you obtain hormones? (*n* = 6)	
Black market	1 (17
Healthcare provider	1 (17)
Internet	3 (50)
Peer/friend	1 (16)
Planning to take hormone therapy in the future	
Yes	32 (74)
No	11 (26)
Missing	7
HIV status	
Negative	47 (94)
Positive	2 (4)
Missing	1 (2)
Hepatitis B infection	
Yes	4 (8)
No	46 (92)
Syphilis	
Yes	3 (6)
No	47 (94)
Pregnant	
No	50 (100)

A minority (9; 18%) had ever used hormone therapy of which six were current users who obtained hormones online (50%), on the black market (17%) or through a healthcare provider (17%). Most (32; 74%) planned to take hormones in the future. The proportion testing positive for HIV, syphilis and HBV was 4%, 6% and 8%, respectively.

### 3.2 Transgender stigma experiences

**Figure 1:**
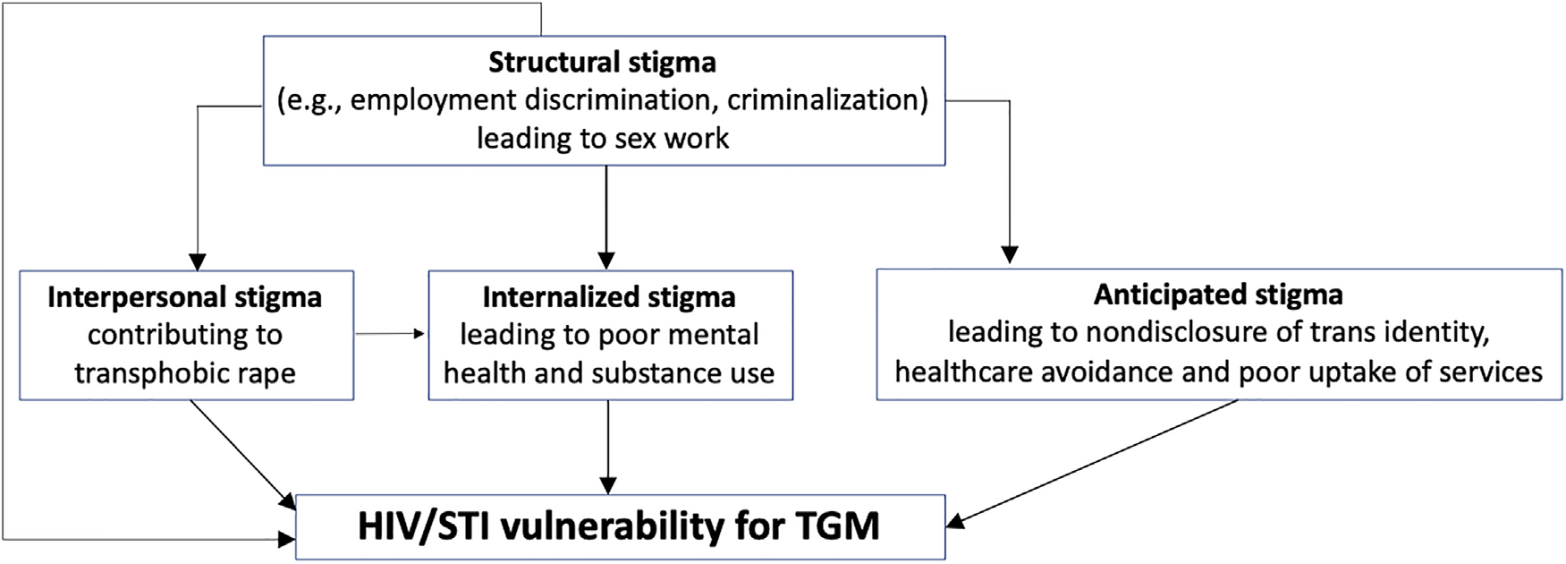
Intersecting stigmas and HIV/STI vulnerability among transgender men

We present four content categories representing the results of the qualitative analysis. Each category describes a different way in which stigma was experienced by trans men in Uganda (Figure 1). The first category describes how their experiences of rape (motivated by transphobic attitudes) led to internalized stigma and shame. The second shows how structural stigma results in economic marginalization and sex work. The third explains the intersection between individual (internalized and/or anticipated) stigma and disclosure. The fourth shows how interpersonal and structural stigma lead to inadequate healthcare. Overall, the lived experiences of trans men highlight the intertwined relationship between stigma and sexual health. Those who experience stigma are marginalized and made more vulnerable to HIV/STIs, while those who are vulnerable to HIV/STIs are less likely to access healthcare because of stigma and discrimination.

### 3.3 Category 1: shame and stigma from transphobic rape

Trans men in this qualitative sample experienced sexual violence, or the threat of it. They were raped by cisgender men “who want to prove a point that you are not a man; you should be a woman.” Transphobic rape was perpetrated to allegedly “correct” gender identity and “teach” trans men how to be cisgender women. The shame and internalized stigma associated with rape made it difficult to talk about being sexually assaulted. The psychological trauma of rape was particularly corrosive to trans male identity. Trans men living with HIV reported that it was difficult to adhere to HIV treatment because taking pills reminded the survivor how they acquired HIV.

“I got HIV through rape. It is something that I have [not] gotten over because I have never talked about it. At times medicine becomes difficult for me because of the thoughts I get about that incident…for someone who has been raped…there is so much in me that has been damaged since I identify myself as a man. While you will say it happens to every woman, but it is more damaging to me” (age 32).

Participants reported that sexual assault was dismissed by family members who prioritized adherence to societal norms over individual self‐expression, and perceived gender non‐conformity as a spiritual problem. The lack of empathy from loved ones following sexual assault further traumatized rape survivors. They were also denied access to post‐rape care services, which compromised their mental and sexual health and perpetuated stigma.

“When I was 13, I was sent to the market in the evening…and when I was coming back, I was raped. I went back home, and I told my grandparent, ‘I have been raped and blood is coming out of me’. She did not care and only told me, ‘You climb so many trees, you ride bicycles like men, it's good you have been raped’. I cannot forget that statement and it hurt so much that they did not care. I was tortured psychologically. I was in pain, and I even started rotting as I had not got treatment…my grandmother had refused to take me to the hospital because I behave in a certain way and that I am possessed by the devil” (age 30).

### 3.4 Category 2: stigma leading to economic vulnerability and sex work

Structural stigma enacted through employer discrimination was experienced before getting a job –– “*You will not get a job where your identity card reads a ‘she’ and they call you a ‘he’*.” It was also experienced after getting employed –– “*Once they got to know [who] I was, I would lose the job*.” Economic vulnerability resulting from lack of employment opportunities and limited educational attainment led trans men into selling sex for survival, which exposed them to the risk of HIV and other STIs. Stigma affected trans men economically, through financial pressure to make ends meet, and socially through the clandestine nature of sex work, which was not disclosed to intimate partners. Poverty, enacted stigma and transgender inequality contributed to engagement in sex work and susceptibility to HIV.

“Some of us have multiple partners and most HIV risks are a result of [our] socio‐economic setup. We are doing sex work because many of us have not finished school so we do not have jobs and the best thing we can do is survive which is through sex work and we are in relationships with people who expect us to take care of them. You find someone doing sex work with a man and they are not telling their partner and there are risks involved because they are having sex with a man for money. They are having sex with their partner for intimacy, and they are not mentioning that they are actually having sex for money outside the relationship” (age 32).

Avoiding HIV infection was not an immediate priority for participants if exchanging sex for money helped to solve pressing financial problems. A hidden population with limited employment opportunities had to find ways to survive economically. The end was perceived to justify the means.

“Me to sleep with my boss when he is giving me two hundred thousand shillings [$56.20] and it can help me solve my problem, at the end of the day, what am I losing? I do it. You don't think of HIV and other diseases. You think about them after solving your problem” (age 29).

### 3.5 Category 3: stigma after disclosure of gender identity or HIV status

Despite these sexual practices, trans men alluded to the common belief that they were not at risk of HIV infection because of non‐penetrative consensual sex with cisgender women. HIV stigma coupled with the stigma of being transgender was perceived to hinder the uptake of HIV testing services and status disclosure.

“That myth that trans men cannot get HIV is common because people believe that the risk of getting HIV is low since vaginal sex is not penetrative. That myth of them not being at risk of getting HIV stops them from testing for HIV, and you do not want to be the only trans man that is HIV positive! It is already too much work [to be] a trans person. People are afraid of knowing of their status…the stigma is still much” (age 25).

Trans men preferred to conceal their identity because “trans stigma that is out there” led to fear of disclosure. Disclosure of gender identity to family members was fraught with difficulty and risked ostracization. It was occasioned by significant events like arrest and incarceration, which necessitated notifying next of kin. Such disclosure had significant consequences, like being asked to leave the family home after “coming out of the closet,” because of stigma based on gender identity.

“When I was arrested [at a club party], I told [my friend] that you know what, ‘My mum is going to get to know that am a gay’. She told me that, ‘You just tell your mum point blank that you are a trans man’. I told her that I cannot tell her such things. She will instead hang me. She insisted that I should tell her… I got my phone and called her while crying. I told her that the truth is, ‘I fall in love with fellow women, but I ask for your forgiveness. That is what I feel like I want’. She was like, ‘You know what, you are not going to stay here. You might spoil my other children. Don't come back here to spoil my children because I gave birth to girl children only’. I got my belongings and left. She told me goodbye. Up to now, we don't communicate” (age 21).

### 3.6 Category 4: healthcare stigma leading to reduced access to services

Trans men avoided public health facilities for fear of how they would be perceived and treated by healthcare workers, preferring instead to receive healthcare from trans‐friendly facilities, such as drop‐in centres. These service delivery points offer walk‐in services, are staffed by members of key populations or friendly healthcare providers and are safe spaces where trans men felt comfortable knowing they would not be asked uncomfortable personal questions.

“How is the public going to look at me? Yeah, I have just entered [a] public hospital; people are staring, people are looking. People want to ask questions so that uncomfortable [feeling] is what I don't want. The trans community is still, there is still that reluctance of going there [health facility]. I think most of them now prefer when a [transgender] organization has a drop‐in center (DIC). They feel a sense of belonging. I know I will be safe there. No one will ask these personal questions so I will gladly want a DIC not a health facility” (age 27).

Healthcare stigma made it particularly difficult to seek care in facilities where they worried about being stigmatized and where it would be difficult to explain that a man had been sexually assaulted. It was difficult for trans men to articulate their vulnerability to healthcare providers. Survivors of sexual violence preferred instead to ask trusted people within the trans community for help with accessing testing and treatment services.

“Trans men [are] not going to tell you that [they] had a sexual encounter with this person but then it ended up as rape. They usually never want to open up and say those things to any health facilities or providers. So, there is going to be that barrier of he has raped me and how do I go for screening? What has he given to me? Probably he had a few infections. I just come in secrecy; I confide in [someone] in the [trans] community that has access to these services. I will not tell them what is wrong, I will just tell them…help me get a [HIV] testing kit and some PEP [post‐exposure prophylaxis]” (age 27).

## 4 DISCUSSION

In this multi‐method study of young trans men in Uganda, stigma was experienced at individual, interpersonal and structural levels. Rape motivated by transphobic beliefs resulted in poor mental health and internalized stigma among rape survivors exacerbating HIV/STI vulnerability. Structural stigma enacted through employment discrimination, limited opportunities for gainful employment and resulted in sexual risk practices and engagement in sex work. Anticipated stigma resulted in fear of disclosure of gender identity and HIV status, poor uptake of HIV services and healthcare avoidance. Overall, stigma operated at multiple intersecting levels to negatively impact health.

Trans men in our study experienced rape that was motivated by transphobic beliefs –– enacted forms of stigma, including physical and sexual violence, due to an individual's gender identity or expression [36]. Sexual violence was perpetrated by cisgender males who used sexual force as punishment for perceived transgression of gender norms [37]. Participants perceived that the perpetrators of “corrective rape” believed it would change transgender identity to cisgender heterosexual identity [38]. Trans men are more likely to be victims of sexual and/or physical violence than the general population [39]. In a study of 284 trans men in nine African countries, the lifetime prevalence of sexual violence was 41% and in the past year 24% [40]. Qualitative research in South Africa has described sexual violence, or the threat of violence, as an ever present reality experienced by trans men [41]. Research in the United States suggests that up to 66% of trans men who are survivors of rape experience repeat sexual assault [42]. A history of sexual assault is associated with adverse mental health outcomes, including substance abuse and suicidal behaviour [43, 44]. Mental illness stigma reinforces the stigma associated with sexual assault and transgender identity [45]. Violence against sexual and gender minorities is a key public health issue [46], requiring gender‐responsive trauma‐informed care [47]. Future studies should evaluate the synergistic interactions between stigma, sexual violence, HIV, and socio‐economic and structural adversity (syndemic vulnerability) among trans men in sub‐Saharan Africa [48].

Structural stigma –– cultural and societal norms, policies and laws that limit opportunities and resources available to stigmatized people –– reduces trans men's access to formal employment and financial services; lack of alternative opportunities drives trans men into sex work, which increases their vulnerability to HIV and other STIs [36, 49]. Trans men enrolled in this study experienced marginalization and economic vulnerability due to limited education and employment opportunities and socio‐economic exclusion, which drove them into sex work. Trans men sex workers find street‐based sex work problematic because of fear of physical violence, rape and murder [50]. They avoid seeking help from the police after experiencing violence for fear of arrest. As a result, online sex work is preferred to working on the street or in brothels. Additionally, condomless sex is more lucrative than sex with a condom further exacerbating HIV vulnerability for an economically marginalized community [51]. In social systems that favour masculinity over femininity [52], trans men who have socially and medically transitioned may or may not be socially perceived as cisgender men. They experience employment discrimination and economic stressors and risk being outed [53]. Structural interventions for economic strengthening should be implemented as part of combination HIV prevention approaches. However, the literature on economic empowerment for trans men is limited, with no data for sub‐Saharan Africa to our knowledge.

Both internalized stigma (negative beliefs a person holds about themselves and their identity) and anticipated stigma (concerns about reactions or behaviours of others were their stigmatized identity to become known) can influence behaviour [36]. In this study, trans men described being afraid to disclose gender identity or HIV status, preferring to remain silent as a form of stigma management. Code switching between identifying as a woman and a trans man was part of their reality depending on the safety of their circumstances [54]. For trans people in sub‐Saharan Africa, disclosure of gender identity and/or sexual orientation or HIV status are associated with a two‐fold increase in lifetime experience of violence [40]. We found that trans men preferred to access care from trans‐friendly providers to avoid stigma and discrimination at public health facilities. Stigmatized persons avoid seeking healthcare because they are mistreated, nervously anticipate rejection (hypervigilance) and are conscious about being rejected (anticipated stigma), leading to delays which exacerbate otherwise preventable diseases [5]. Stigma and social exclusion affect self‐esteem and self‐worth, and contribute to anxiety, depression, self‐harm and substance abuse, factors associated with HIV and STI vulnerability [5, 14].

Healthcare stigma operates at interpersonal and structural levels and is rooted in the ways that society negatively views trans men [36]. Lack of provider knowledge about the health needs of trans men, discrimination and implicit bias are significant barriers to sexual health services [55]. Mistreatment of trans people in healthcare settings is common and leads to the avoidance of subsequent healthcare interactions [5, 56]. Qualitative studies have shown that lack of healthcare provider training causes discomfort for providers and patients [5]. Providers in sub‐Saharan Africa receive little or no training in trans‐friendly care. However, provision of gender sensitivity training to providers may improve sexual health services [57], and is a recommended intervention to mitigate healthcare stigma [36].

We found that HIV prevalence in our study of trans men was lower than that reported among cisgender women in the 2017 Uganda Population‐Based HIV Impact Survey (7.6%) [58]. HIV prevalence among trans men in Uganda is unknown. Although we recruited trans men irrespective of HIV status, snowball sampling may have biased the sample towards individuals without HIV. By contrast, HBV prevalence in our study (8.0%) was twice as high as the general population (4.1%), reflecting unmet sexual health needs among trans men. Routine HBV vaccination is recommended for individuals with risk factors for HBV infection, such as condomless anal or vaginal sex with multiple partners or with a partner with HBV infection [59], and should be offered to trans men in this setting. Future studies of trans men will help to characterize STI risk in greater detail.

Our study strengths include the successful enrolment of a novel sample of trans men who are largely hidden and invisible to HIV programming and research in sub‐Saharan Africa. Our study adds to the knowledge base of HIV and STI risk and stigma experiences of trans men in sub‐Saharan Africa. The limitations of this exploratory work include the cross‐sectional design. Non‐random sampling may have led to the selection of trans men with greater HIV/STI vulnerability. The inability to determine temporal relationships between outcomes and risk factors limited the analysis of behavioural correlates of HIV/STI infection. We had limited funding for broader STI testing and did not test for other curable STIs. The small sample size may not be representative of trans men in Uganda. Our results, obtained from one research clinic, are not generalizable but may inform programmatic delivery of sexual health services for this population in Uganda and potentially other African settings. Nevertheless, our study contributes to a scarce body of literature on stigma and HIV vulnerability among trans men in sub‐Saharan Africa.

## 5 CONCLUSIONS

Our findings underscore the urgent need for trans men to access safe and friendly comprehensive HIV/STI services that are tailored to their specific needs. This need is underscored in settings where HIV and other STIs are concentrated among key populations, unemployment is rife, sex work is illegal and healthcare access is limited. Trans men exposed to sexual violence should be provided with gender sensitive and respectful survivor‐centred support, and access to justice and legal redress. Multi‐level interventions are needed to address trans stigma at individual, interpersonal and structural levels and improve health outcomes.

## COMPETING INTERESTS

AM reports a grant from Gilead Sciences, Inc outside the submitted work, and was an advisor to ViiV Healthcare. JEH is a consultant for Merck. JMB works for Gilead Sciences, Inc. but was faculty at the University of Washington, Seattle, USA at the time of the study.

## AUTHOR CONTRIBUTIONS

AM, JEH and NCW conceptualized the manuscript. MSN conducted the statistical analyses. VK, BK, AN, JM and CCT conducted qualitative interviews and transcription. MAW, TRM, JEH and AM supervised qualitative data collection and study implementation. AM and VK coded the data. AM wrote the first draft along with JEH. ACT, TRM, JEH, MAW, JMB and NCW reviewed drafts and provided substantial edits. All authors read and approved the final version.

## FUNDING

This work was supported through research grants from the United States National Institutes of Health (grants K43 TW010695 and AI027757 [AM] and K24 mentor award MH114732 [JEH]). Study data were collected using REDCap, funded through grants UL1 TR002319, KL2 TR002317 and TL1 TR002318 from the National Center for Advancing Translational Sciences. This paper represents the opinions of the authors and does not necessarily represent the official views of the National Institutes of Health.

## Data Availability

Access to databases generated under the project will be available for educational, research and non‐profit purposes as approved by the relevant IRB's.
